# Temperature-Dependent Mechanical and Structural Properties of Uniaxially Strained Planar Graphene

**DOI:** 10.3390/ma18225179

**Published:** 2025-11-14

**Authors:** Sané Erasmus, Charalampos Skokos, George Kalosakas

**Affiliations:** 1 Nonlinear Dynamics and Chaos Group, Department of Mathematics and Applied Mathematics, University of Cape Town, Rondebosch 7700, South Africaharis.skokos@uct.ac.za (C.S.); 2 Max Planck Institute for the Physics of Complex Systems, Nöthnitzer Str. 38, D-01187 Dresden, Germany; 3 Department of Materials Science, University of Patras, GR-26504 Rio, Greece

**Keywords:** graphene, molecular dynamics, stress-strain response, elastic properties, bond length and angle distributions

## Abstract

Using molecular dynamics simulations of a planar graphene sheet, we investigate the temperature dependence of its mechanical behavior under uniaxial tensile stress applied either in the armchair or zigzag direction. Stress–strain curves are calculated for different temperatures, and the corresponding dependence of various elastic parameters is discussed. Fracture stress and strain, as well as the Young’s modulus, decrease almost linearly with temperature, in accordance with previous investigations. An almost linear variation in the third-order elastic modulus with temperature is demonstrated, revealing opposite trends for uniaxial loadings in the armchair or zigzag direction. The detailed dependence of the distributions of bond lengths and bond angles both on strain and temperature is presented for the first time, along with approximate analytical expressions. The latter accurately describe the numerically obtained distributions.

## 1. Introduction

Since the discovery of graphene, there have been a number of investigations into its mechanical behavior. Despite the difficulty of applying controlled mechanical loads at the nanoscale, experimental studies have verified an exceptional value of stiffness and extremely high tensile strength [1,2,3], in accordance with corresponding theoretical predictions. For example, in Reference [1], the mechanical properties of graphene were deduced from nanoindentation experiments conducted on circularly clamped samples, while in References [2,3], push-to-pull testing was employed to directly apply uniaxial strain. There exist a number of related numerical investigations using molecular dynamics (MD) simulations with a variety of potential functions [4,5,6,7,8,9,10,11,12,13,14,15,16,17,18,19,20,21,22,23,24,25,26], density functional theory [27,28,29,30,31,32,33], or other theoretical approaches, including molecular mechanics [34,35,36] and combinations of continuum elasticity theory with other methods [37,38,39,40].

The temperature dependence of the various elastic properties of graphene has been examined using MD [7,9,20], Monte Carlo atomistic simulations [4], density functional theory [33], and the asymptotic homogenization method [41]. Moreover, a few MD studies have investigated the variation in bond lengths and bond angles with uniaxial tensile loading [5,42]. The latter work showed results obtained using first-principles methods too and presented analytical expressions for the dependence of these structural parameters on strain [42]. The variation in bond lengths and angles with biaxial strain has been discussed in [43]. To our knowledge, the dependence of the distributions of bond lengths and bond angles on both temperature and strain has not yet been examined.

In this work, we used MD simulations to study the behavior of planar graphene under uniaxial tensile load, considering the influence of temperature. In particular, we implemented symplectic integration methods for simulating the system’s time evolution, allowing highly accurate computations for arbitrarily long times. We calculate stress–strain curves at various temperatures, and from these results, we estimated the variation in several elastic parameters with temperature. Our findings for the Young’s modulus, fracture strength, and failure strain are in agreement with previous studies. The temperature dependence of the the third-order elastic modulus has not been reported up to now.

Furthermore, we compute bond lengths and bond angles of bulk graphene over a large time-window after thermal equilibrium has been reached and subsequently analyze these results in order to determine the dependence of the corresponding distributions on both stress and temperature. Finally, we present analytical expressions that closely match the numerically obtained distributions of bond lengths and angles. Thus, we describe—for the first time, to the best of our knowledge—the detailed dependence of these structural properties of graphene on both the applied tensile stress and temperature.

This paper is organized as follows. In Section 2, we present the force field used, along with the numerical methods we implemented. The results of our investigation are discussed in Section 3. In particular, the implementation of finite temperatures in our MD microcanonical simulations is discussed in Section 3.1, and thermal effects on graphene’s mechanical response under uniaxial tension are studied in Section 3.2. Then, we present the distribution of bond lengths and bond angles in the planar sheet of $sp^{2}$ carbon atoms under various stresses and at various temperatures in Section 3.3, while analytical expressions for said distributions are determined in Section 3.3.1. Finally, we conclude our findings in Section 4.

## 2. Model and Numerical Methods

**Figure 1:**
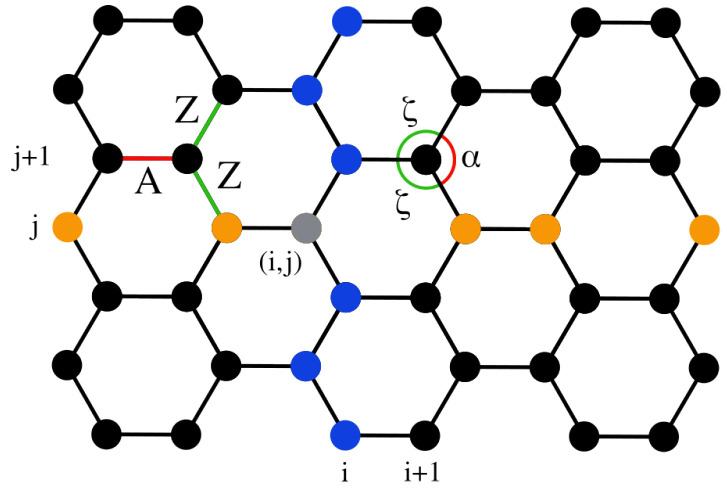
A schematic of the hexagonal graphene lattice depicting N=42 atoms, arranged in $N_{I}=6$ columns and $N_{J}=7$ rows. Atoms in column *i* and row *j* are indicated in blue and orange, respectively, and the (i,j)th atom is in gray. The *A*- and *Z*-type bonds, and similarly the α- and ζ-type angles, are indicated in red and green, respectively (see Section 3.3 for more details on these distinctions).

We consider a two-dimensional (2D) model of graphene as a hexagonal lattice of carbon atoms within a plane. Figure 1 illustrates a part of this structure at equilibrium, where the distance between any two neighboring atoms is $r_{0}=1.42$ Å, and the angle formed by three consecutive atoms is $\phi_{0}=2\pi /3$ rad. Furthermore, all carbon atoms have mass m=12 amu. In the orientation depicted in Figure 1, the top and bottom edges represent the “armchair edges”, while the left and right edges correspond to the “zigzag edges”. Furthermore, it is common to call the “armchair direction” the horizontal direction (as shown in Figure 1) and the “zigzag direction” the vertical one.

We refer to columns and rows within the graphene sheet, as indicated by the blue and orange atoms, respectively, in Figure 1. A lattice of size $N=N_{I}\times N_{J}$ consists of $N_{I}$ columns, which are indexed by *i*, and $N_{J}$ rows, which are indexed by *j*. The (i,j)th atom is indicated in gray in Figure 1. Thus, the configuration shown in this figure corresponds to a $N_{I}\times N_{J}=6\times 7$ lattice, totaling N=42 atoms. In our simulations, we consider a lattice of $N_{I}=86$ columns and $N_{J}=87$ rows of atoms, resulting in a N=86×87=7482 total number of carbon atoms. This lattice size is sufficiently large to negate small-size effects [12], allowing us to represent the behavior of bulk graphene while still permitting extensive numerical simulations within reasonable CPU times. We have further confirmed that indicative data for larger lattices are similar to the ones presented here.

A Hamiltonian formalism is used to investigate the in-plane dynamics of the lattice in a similar fashion as in Reference [44]. The atomistic force field, describing bond-stretching and angle-bending deformations, has been determined through fittings with relevant density functional energy computations [12]. In particular, the potential energy of a covalent bond between nearest neighboring carbon atoms at distance *r* is given by the Morse expression,

(1) $$V_{M}\left(r\right)=D{\left(e^{-a\left(r-r_{0}\right)}-1\right)}^{2},$$

where D=5.7 eV is the depth of the potential well and a=1.96 Å^−1^ is the inverse characteristic length scale of the potential. The angle-bending energy term describing a bond angle ϕ formed by three consecutive atoms is

(2) $$V_{B}\left(\phi \right)=\frac{d}{2}{\left(\phi -\phi_{0}\right)}^{2}-\frac{d^{\prime }}{3}{\left(\phi -\phi_{0}\right)}^{3},$$

where d=7.0 eV/rad^2^ and $d^{\prime }=4$ eV/rad^3^ are the constants of the quadratic and nonlinear term of the potential, respectively.

A limitation of the force field used, given by Equations (1) and (2), is the decoupling of the bond-stretching and angle-bending variables. Though this is a standard approximation in atomistic simulations, in real systems, these degrees of freedom are coupled. However, the predictions of this model for the Young’s modulus and the intrinsic strength of graphene are in good agreement with the experimental estimates of Reference [1], while the stress–strain response is in accordance with corresponding calculations from first principles [12]. A more drastic approximation is that the out-of-plane atomic displacements are ignored. At finite temperatures, ripples and other out-of-plane deformations spontaneously appear in graphene due to thermal fluctuations [45,46]. However, we expect that at relatively low temperatures, these non-planar deformations would be suppressed when graphene is uniaxially stressed. Stretching has been experimentally used to flatten graphene [2], and this has been further confirmed by MD simulations [47]. Thus, we have considered here only temperatures up to 700 K and not higher ones, even though graphene exists at much higher temperatures. As will be discussed later, the intrinsic strength and fracture strain obtained via our model at room temperature are in good agreement with those obtained from fully three-dimensional MD simulations employing different interatomic potentials [3,5,47].

The total energy of the system (i.e., the values of the model’s Hamiltonian *H*) is the sum of the above potential energy terms for all bond lengths between nearest neighboring atoms and all bond angles between adjacent bonds and the kinetic energy of each atom. Denoting the total potential energy at time *t* by $E_{V}\left(t\right)$ and the total kinetic energy at *t* by $E_{K}\left(t\right)$, the Hamiltonian

(3) $$H=E_{K}\left(t\right)+E_{V}\left(t\right),$$

is expressed through the positions (x(t),y(t)) and the corresponding conjugate momenta of all carbon atoms within the considered graphene sheet. The time evolution of each atom’s position and momentum is governed by the system’s Hamilton’s equations of motion, which conserve the total energy, Equation (3).

To investigate the effects of uniaxial tensile load, a constant force *f* is applied to all atoms on the appropriate edges of the sheet [12,42]: For stress/strain in the armchair direction, the force *f* is applied on the atoms of the zigzag edges, where, on the opposite edges, opposite forces, directed outwards, are applied. Similarly, for stress/strain in the zigzag direction, the force *f* is applied to the armchair edges, again with opposite forces on opposite edges. Tensile loading results in additional terms in the Hamiltonian, given by appropriate products of the relevant edge atom displacements with the applied force *f*. For constant forces, as in our case here, the conservation of the system’s total energy still holds.

We must emphasize that we perform stress-controlled simulations here, where we fix the forces (stress) and directly compute the resulting strain. This is a natural choice in MD, in contrast to imposing fixed displacements (strain-controlled simulations), which are preferred in first-principles studies. In principle, these two methods of strain- or stress-controlled simulations are equivalent for estimating the stress–strain mechanical response of the system. For example, one can see in Figures 4 and 5 of Reference [12] a direct comparison of stress-controlled MD data and strain-controlled density functional theory data, which produce identical results, at least in the linear response regime.

In two-dimensional materials like graphene, the stress is expressed as force per unit length. Taking into account the distance between successive atoms at the relevant edges where the force is applied, i.e., the nearest neighboring atoms along an edge column, or row, in Figure 1, concerning stress in the armchair or zigzag direction, respectively, the following relations connect nominal stresses and forces

(4) $$\sigma_{a}=\frac{f}{r_{0}\sin\left(\phi_{0}/2\right)}\;\;\;\;\mathrm{and}\;\;\;\;\sigma_{z}=\frac{f}{0.5\;r_{0}\left[1+\cos(\phi_{0}/2)\right]},$$

where the indices *a* and *z* denote stress in the armchair and zigzag direction, respectively, while $r_{0}$ and $\phi_{0}$ are the equilibrium values mentioned above.

To determine, at a temperature of zero, the relaxed state of the strained graphene for various applied stresses in any direction, a friction term proportional to the velocity of each atom is incorporated in the MD simulations, with the friction coefficient set to γ=0.1 ps^−1^; see Reference [12] and the discussion therein. Then, the fourth order Runge-Kutta numerical integration technique is used with an integration time step of $dt=0.005\;t_{u}$, where $t_{u}=0.0102$ ps represents the time unit in our model. This time step ensures that the relative energy error $Err\left(t\right)=\left|H(t)-H(0)\right|/\left|H(0)\right|$ is below $10^{-7}$ in corresponding energy-conserving simulations where the friction term is absent. However, we now simulate the dynamics of the dissipative version of the system until times $t_{f}=3\times 10^{3}\;t_{u}$, when the total kinetic energy is practically zero ($E_{K}\left(t_{f}\right)<10^{-16}$ eV). In this way, we determine the relaxed equilibrium positions of the atoms within the lattice for each considered value of stress σ.

Based on this equilibrium configuration of graphene subjected to tensile loads, we embark on the main phase of the numerical investigation: following the dynamics of the lattice for a fixed value of stress σ at various temperatures *T* for a long enough time to allow the formulation of deductions about the thermal-equilibrium properties of the stressed material. For these numerical simulations, we implement the symplectic integrator ABA864 [48], with an integration time step $dt=0.06\;t_{u}$, which results in relative errors of the total energy $Err\left(t\right)<10^{-7}$ for all times. This particular integration scheme has been shown to perform very well in balancing computational speed and numerical accuracy for multidimensional Hamiltonian lattices [49] and was successfully used for examining chaos in graphene [44].

The relaxed equilibrium positions that have previously been determined for the given value of stress σ correspond to a graphene sheet being at zero temperature, without thermal fluctuations. In order to simulate the system at finite temperatures, following a suitable energy–temperature calibration (see Section 3.1 below), we randomly insert an additional energy density (average energy per site) $e_{N}$ on the relaxed T=0 K state. This additional energy is initially provided as solely potential energy, in the form of small random displacements of each atom from the relaxed zero-temperature positions. Then, these displacements are properly scaled in order to adjust the added energy density $e_{N}$ to the desired value corresponding to the simulated temperature. During evolution, the initial potential energy is shared as kinetic and potential energy, and eventually the system equilibrates.

In general, for the numerical results presented in the next section, we consider 10–20 different individual realizations of the randomly added initial energy, but we have selectively checked the robustness of the data when more realizations are used. For each realization, we calculate the temporal evolution of the various quantities of interest and then compute the average of these time series over the different realizations in order to determine the time dependence of the considered quantities for the ensemble. We denote such an averaged quantity over the different realizations with angled brackets, e.g., $\langle M(t)\rangle$ for the measurement of the quantity M(t). We may further determine the average of a thermally equilibrated quantity over time. In such a case, we average both over initial realizations and over time intervals, and we denote the computed average by using both an overline and angled brackets, e.g., $\bar{\langle M\rangle }$ for a variable *M* at thermal equilibrium.

At finite temperatures, the size of graphene sheets exhibits oscillations around their T=0 K relaxed configurations due to the thermal energy of the system (discussed further in Section 3.2 below). In order to collect data over enough of such sheet oscillations, we follow the system’s time evolution up to $4\times 10^{3}\;t_{u}$. The recording window for all subsequent measurements is from $1\times 10^{3}$ to $4\times 10^{3}\;t_{u}$, totaling $3000\;t_{u}$. We checked the insensitivity of the obtained results to the length of the recording window by testing longer time windows.

## 3. Results and Discussion

### 3.1. Temperature Calibration

When an energy density $e_{N}$ is inserted into the strained graphene lattice, we observe that, initially, the total kinetic energy increases with time from its starting value of zero; then, following some relatively large fluctuations, the system settles at thermal equilibrium after at most $10^{3}\;t_{u}$. The time evolution of the system’s temperature T(t) towards equilibrium is computed in our microcanonical MD simulations through the energy equipartition relation

(5) $$T\left(t\right)=\frac{E_{K}\left(t\right)}{N\;k_{B}}$$

where $k_{B}$ is the Boltzmann constant.

In order to test whether thermal equilibrium has been reached, we compare the mean value and the standard deviation of the fluctuating temperature over various time windows. Before thermal equilibrium is achieved, the standard deviation of the time-averaged *T* is relatively large, and it also changes depending on the time window. When thermal equilibrium is reached, the temperature fluctuations and the standard deviation are consistently small. The mean temperature at thermal equilibrium is calculated by averaging over both the individual realizations and the recording time-window. We denote this average value $\bar{\langle T\rangle }$ as $T_{ave}$. In this case, the standard deviation of the measured values is computed using all data points over realizations and time.

**Figure 2:**
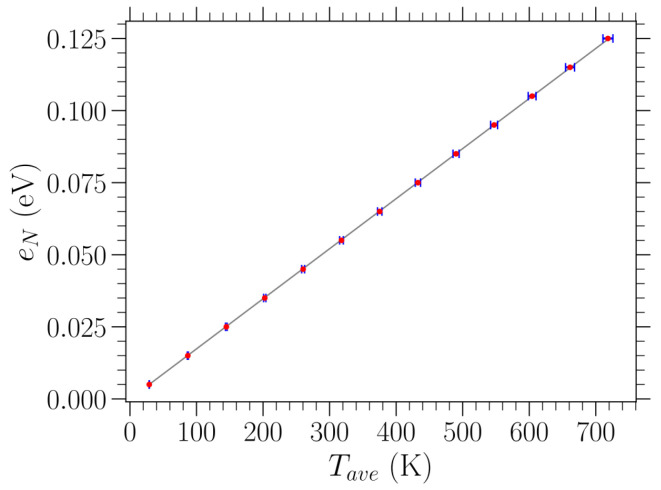
Red dots represent the relation between the additional energy density $e_{N}$ above the relaxed T=0 K graphene structure subjected to uniaxial tensile stress σ=2.16 eV/Å^2^ in the zigzag direction and the average temperature $T_{ave}$ at thermal equilibrium, evaluated through the MD simulations by averaging over both time and the different realizations. One standard deviation of the $T_{ave}$ measurements is indicated by blue horizontal error bars. The linear fitting of the presented data points is indicated by the gray solid line, providing a slope equal to $1.74\times 10^{-4}$ eV/K.

The relationship between the additional energy density $e_{N}$ on top of the relaxed equilibrium loaded structures and the averaged temperature $T_{ave}$ is linear in all cases of different stresses examined here, at least for temperatures up to 700 K, as considered in this work. One representative case is shown in Figure 2. The resulting slopes from the linear fittings of the data are very close for all values of stress σ (a difference in the computed values is observed only in the fourth significant digit), and they are slightly above $2k_{B}$ due to the nonlinearities of the potential energy. For finite loads, the slope slightly increases with the amount of stress. Thus, for a given value of stress σ, we use the obtained slope of the $e_{N}$ versus $T_{ave}$ linear fitting in order to control temperature (within a 1% accuracy) in our investigation. In particular, we set the amount of the added energy density $e_{N}$ according to the desired temperature value.

In order to investigate the system’s elastic and structural properties, as discussed in the following sections, we collected data from the central region of the lattice to avoid potential edge effects and thus represent the behavior of bulk graphene. In particular, this central region sub-lattice has a geometry analogous to the larger structure, with $n_{I}=44$ columns and $n_{J}=45$ rows.

### 3.2. Mechanical Response

In the stress-controlled numerical implementation used here to examine the mechanical response of graphene, we compute the resulting strain due to the fixed force applied at the appropriate edge atoms. The uniaxial strain ϵ is obtained through the strain of the central row of the graphene sheet when the stress is applied in the armchair direction, while it is calculated by the average strain on the two central columns of the sheet in case of stress in the zigzag direction (see Figure 1). $\epsilon_{T}$ indicates the strain corresponding to temperature *T*.

In the zero-temperature case, i.e., where T=0 K, the uniaxial strain $\epsilon_{0}$ is determined through the relative change in the length of the central row (two central columns) of graphene subjected to a given applied stress in the armchair (zigzag) direction with respect to the length of the central row (columns) in the unstrained equilibrium configuration shown in Figure 1. These measurements are taken in the central region of the lattice, as mentioned at the end of the previous subsection. For any length computations discussed here, a horizontal (vertical) length is measured as the difference of the *x* (*y*) coordinates of the edge atoms considered. The stress–strain response is obtained in this way at 0 K.

When the temperature of the system is raised at finite values, by adding energy to the equilibrated graphene, the lattice stretches and compresses in an oscillatory manner. The details of these oscillations depend on the temperature and the applied stress and will be investigated in the future. In this case, one has to take into account that the strain measurement $\epsilon_{T}\left(t\right)$ is now exhibiting temporal oscillations. Since we consider 10-20 different realizations of the randomly inserted initial energy distribution, we register the average, over these realizations, strain in time $\langle \epsilon_{T}\left(t\right)\rangle$, noting that the aforementioned oscillations are in-phase in the different realizations.

For evaluating the strain $\epsilon_{T}$ of a uniaxially loaded graphene sheet at finite temperatures, a reference length $\ell_{T}^{ref}$ corresponding to zero stress σ=0 at the particular value of *T* is needed. This reference length accounts for thermal effects on the initial configuration, and it is obtained by calculating the average, over realizations and time, of the length of the central row, or columns, of the sheet in the absence of a load. Then, when a stress is applied, the time evolution of strain in a particular realization is determined as the relative change in the length with respect to the reference length $\ell_{T}^{ref}$

(6) $$\epsilon_{T}\left(t\right)=\frac{\ell \left(t\right)-\ell_{T}^{ref}}{\ell_{T}^{ref}},$$

where ℓ(t) is the length at time *t* of the central row or the average length of the two central columns depending on the direction of the applied uniaxial load.

**Figure 3:**
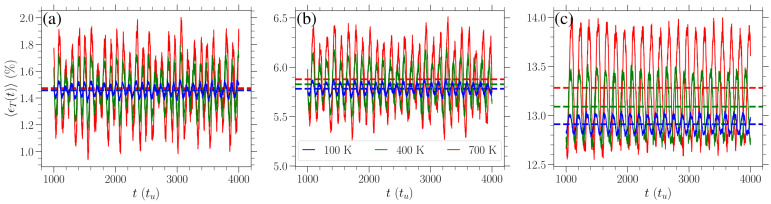
Time evolution of the average (over individual realizations) strain $\langle \epsilon_{T}\left(t\right)\rangle$, Equation (6), when stress—(**a**) σ=0.188 eV/Å^2^, (**b**) σ=1.03 eV/Å^2^, and (**c**) σ=1.97 eV/Å^2^—in the zigzag direction is applied for different temperatures: T=100 K (blue curves), T=400 K (green curves), and T=700 K (red curves). The average (over realizations and time) strains $\bar{\langle \epsilon_{T}\rangle }$, for each temperature, are indicated by the horizontal dashed lines of the same color in each panel.

In Figure 3, we highlight the behavior of $\langle \epsilon_{T}\left(t\right)\rangle$ for various values of stress σ in the zigzag direction at three distinct temperatures *T*, which are indicated by different colors. An increase in temperature leads to an increase in the amplitude of strain oscillations as well as in the average strain. The latter is obtained as the average over both realizations and time, $\bar{\langle \epsilon_{T}\rangle }$, and it is indicated by the dashed horizontal lines of different colors depending on the temperature in Figure 3. The average strains $\bar{\langle \epsilon_{700}\rangle }$ (red horizontal dashed lines) about which the T=700 K curves oscillate are higher than the $\bar{\langle \epsilon_{100}\rangle }$ (blue horizontal dashed lines) of the T=100 K curves in all cases of different stress. However, these differences are larger for absolute values for larger stresses.

We have validated that if one follows an alternative path on the (σ,T) plane by first giving initial energy to the system and then applying forces at the edges, practically identical average strains are obtained. However, our approach is much more efficient because the temperature is accurately controlled and, more importantly, the system reaches thermal equilibrium significantly faster; in the alternative method, the equilibration takes orders of magnitude longer.

**Figure 4:**
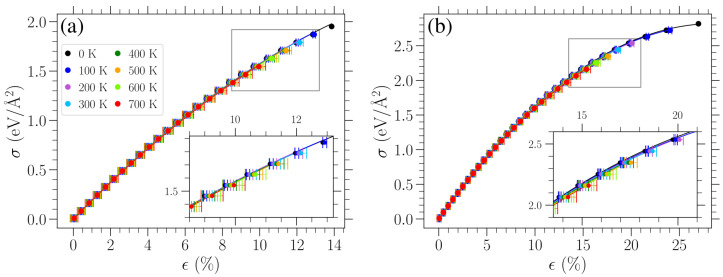
Stress–strain response of planar graphene for uniaxial loads in the (**a**) armchair and (**b**) zigzag directions for different temperatures as indicated in the legend. Filled circles indicate the obtained average strain for each given stress. Solid curves represent fittings of these data with Equation (7) (see text). For T≠0 K, the strain is given as the average over time and realizations, $\bar{\langle \epsilon_{T}\rangle }$, and the error bars correspond to one standard deviation. The insets in each panel depict a close-up view of the region indicated by the gray rectangle in each panel.

Calculating the average strain $\bar{\langle \epsilon_{T}\rangle }$, as mentioned above, allows one to obtain the mechanical response of planar graphene at different temperatures. Stress–strain curves for uniaxial tensile loads in the armchair and zigzag directions are presented for various temperatures in Figure 4. Despite the small differences, one can see for larger stresses that the average strain is a bit further to the right for the higher-temperature cases. The error bars indicate the standard deviation of the average strain measurement. As one can also deduce from Figure 3, the standard deviation is higher for higher temperatures. This is highlighted via the insets in each panel, where a close-up of the data points and error bars is presented for the region, which is indicated by the gray rectangle in each panel. When examined close-up, it is easier to see that the lengths of the error bars increase with temperature.

We have checked the accuracy of the presented strain measurements when more realizations or longer time windows are considered. In particular, the obtained strain values differ in the 3rd significant digit at most when the number of realizations is increased or the length of the time window is doubled.

Since, at finite temperatures, strain is measured with respect to the averaged oscillating length $\ell_{T}^{ref}$ due to thermally induced vibrations of the unstrained sheet, the stress–strain curves pass through the origin of Figure 4, as expected. From Figure 4, we can see that the temperature has a relatively small effect on the stress–strain response, at least for the values of *T* considered here, apart from the significant reduction in the fracture point. For small stresses, the achieved strain is practically the same for the two directions of applied stress, while the strong directional dependence at large stresses has already been well established in previous investigations [5,12,20,28].

**Figure 5:**
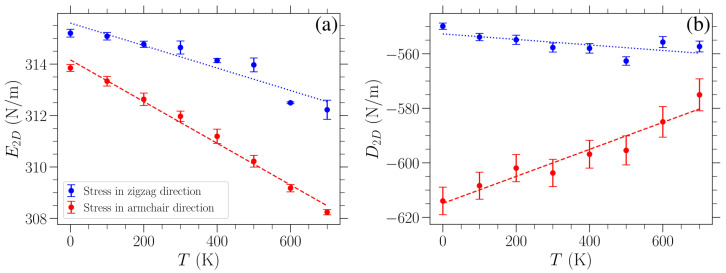
Temperature dependence of (**a**) the Young’s modulus $E_{2D}$ and (**b**) the third-order elastic modulus $D_{2D}$ for applied stress in the armchair (red points) or zigzag (blue points) direction, evaluated through fittings of the data in Figure 4 with Equation (7) (see text).

The stress–strain response can be described by the nonlinear relation [12]

(7) $$\sigma =E_{2D}\;\cdot \;\epsilon +D_{2D}\;\cdot \;\epsilon^{2},$$

where σ is the applied uniaxial stress, ϵ is the corresponding strain, $E_{2D}$ is the 2D Young’s modulus, and $D_{2D}$ is the 2D third-order elastic modulus. For each temperature examined and both directions of applied stress, we first obtained the value of Young’s modulus using the linear response at small stress/strain, and then we fit the data presented in Figure 4 with Equation (7) to determine the third-order elastic modulus. The computed values of $E_{2D}$ and $D_{2D}$ are plotted in Figure 5 as a function of temperature for applied stress in either the armchair (red points) or the zigzag (blue points) direction. The error bars on these points indicate one standard deviation of the fitted parameters under the observed covariance of the fit. A linear variation can roughly approximate the obtained temperature dependence of these elastic moduli. Linear fittings of the corresponding data are indicated by the dashed red (dotted blue) line for stress in the armchair (zigzag) direction.

The Young’s modulus $E_{2D}$ (Figure 5a) appears to decrease almost linearly with an increasing temperature, albeit only by a relatively small amount, which is consistent with other results in the literature [7,33,41]. In particular, the linear fitting of these data for stress in the armchair direction leads to a variation in $E_{2D}\left(T\right)$ with a slope of $-8.1\times 10^{-3}$ (N/m)/K, while for stress in the zigzag direction, the slope is $-4.4\times 10^{-3}$ (N/m)/K.

The decrease in the Young’s modulus with temperature is often given in the literature as a percentage change over the investigated range of temperatures. To ensure we make a superior direct quantitative comparison, we present our results along with existing ones in the literature as percentage change per 100 K. In our case, the Young’s modulus decreases by 0.25%/100 K and 0.13%/100 K for stress in the armchair or the zigzag direction, respectively. Combined density functional theory and quasi-harmonic approximation calculations in Reference [33] yielded a $E_{2D}$ decrease by 0.22%/100 K over the range from 0 K to 1000 K. Molecular dynamics was used in Reference [7] for investigations, at 300 K, 500 K, and 700 K, of graphene lattices of different aspect ratios consisting of 1886 atoms. $E_{2D}$ was found to decrease by 1.3%/100 K, 0.48%/100 K, and 0.33%/100 K (0.88%/100 K, 0.65%/100 K, and 0.33%/100 K) for strain in the armchair (zigzag) direction, where the three different values correspond to graphene aspect ratios of 1.97, 1.44, and 1.01 (1.95, 1.45, and 0.99). We note that our lattice has an aspect ratio of 1.72. Molecular dynamics simulations in Reference [14] resulted in a $E_{2D}$ decrease by 1.4%/100 K over a temperature range of 300 K to 2000 K for strain applied in the armchair direction. Finally, in Reference [41], a reduction in $E_{2D}$ between 0.19%/100 K and 0.25%/100 K was obtained for temperatures ranging from 0 K to 1600 K, where the varying reduction depends on the different parameterizations of the model used, thus affecting the Young’s modulus value at T=0 K.

The calculated Young’s modulus $E_{2D}$ at 300 K is in good agreement with values reported in experimental studies conducted at room temperature [1,2,3]. In particular, our findings, namely, 315 N/m and 312 N/m for loading in the zigzag and armchair directions, respectively, are consistent with the Young’s modulus reported in Reference [1] (340 ± 50 N/m), [2] (300 to 340 N/m), and [3] (350 ± 100 N/m).

We can observe from Figure 5b that the $D_{2D}$ values are consistently higher for strain in the zigzag direction than for the other direction. This is congruent with the fact that the graphene sheet is more resistant to stress in the zigzag direction. When stress is exerted in the armchair direction, one third of all the bonds are parallel to the direction of strain, and hence these bonds exhibit maximal stretching in the sheet. Taking into account the respective angle deformations, there is generally higher strain for the same stress in this case as compared to loads in the zigzag direction. As the Young’s modulus is almost the same in these two cases, there is a lower $D_{2D}$ modulus (i.e., higher absolute values) for stresses in the armchair direction. The different strains for a given stress in the two perpendicular loading directions discussed here can be seen when comparing the panels of Figure 4, where the curves for stress in the armchair direction lie further to the right than when the stress is applied in the zigzag direction, indicating higher strains for the same stress. Regarding the temperature variation of $D_{2D}$, different trends are exhibited when the stress is in the zigzag or armchair direction. A linear fitting of the $D_{2D}\left(T\right)$ data points results in a slope $+5.0\times 10^{-2}$ (N/m)/K for strain in the armchair direction and $-1.0\times 10^{-2}$ (N/m)/K for strain in the zigzag direction. The value of $D_{2D}$ increases by 0.91%/100 K (decreases by 0.19%/100 K) for strain in the armchair (zigzag) direction over the temperature range from 0 K to 700 K.

**Figure 6:**
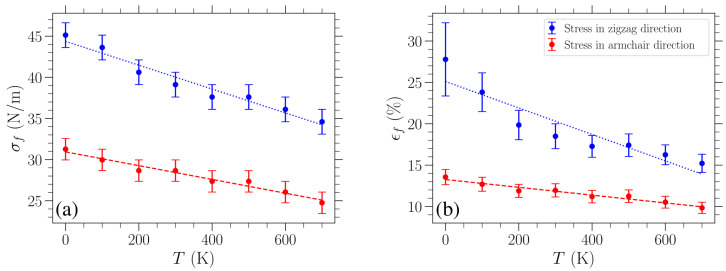
Temperature dependence of (**a**) the fracture strength $\sigma_{f}$ and (**b**) associated failure strain $\epsilon_{f}$ of graphene. Straight lines represent linear fittings.

Finally, we estimate the graphene’s fracture strength $\sigma_{f}$ and failure strain $\epsilon_{f}$ for different temperatures *T*. The former is obtained by using the highest tested value of stress σ that does not lead to failure of the graphene sheet. Its error bar is provided by the step we used when increasing the tested σ values, which are evenly spaced. These results are presented in Figure 6a, where an almost linear decrease in fracture stress with temperature is shown. A linear fitting of these data points is indicated with a dashed red (dotted blue) line for stress in the armchair (zigzag) direction. The slope of the linear fitting of the $\sigma_{f}\left(T\right)$ data is $-8.4\times 10^{-3}$ (N/m)/K for stress in the armchair direction and $-1.5\times 10^{-2}$ (N/m)/K for stress in the zigzag direction.

Such a linear dependence of the fracture strength on temperature is in accordance with existing results. In particular, we estimate that, based on the MD study on graphene loaded in the armchair direction reported in Reference [9], fracture strength decreases with temperature at a rate of $-8.6\times 10^{-3}$ (N/m)/K, which is in very good agreement with the slope obtained in our work for the same direction. Molecular dynamics simulations were also used in Reference [40], and the presented temperature dependence of the fracture stress decreased linearly with a slope of $-1.4\times 10^{-2}$ (N/m)/K. However, the loading direction was unclear in the cited case. In another MD investigation, where the stated loading direction was the armchair direction, the corresponding slope was found to be $-1.6\times 10^{-2}$ (N/m)/K [14]. Lastly, Reference [24] employed a combination of machine learning and MD simulations with the Tersoff potential. In that study, the fracture strength decreased linearly with temperature, exhibiting a slope of $-3.8\times 10^{-2}$ (N/m)/K for loading in the zigzag direction, whereas for loading in the armchair direction, a bilinear behavior is observed: the slope is initially $-6.7\times 10^{-3}$ (N/m)/K but becomes steeper above 500 K, thus representing the only non-linear trend reported in the literature.

The failure strain, $\epsilon_{f}$, at different temperatures has been estimated through the value of fracture stress by solving for $\epsilon_{f}$ in Equation (7). To this end, the known value of $\sigma_{f}$, as well as the fitted values of $E_{2D}$ and $D_{2D}$ describing the stress–strain curve at the given temperature, has been used. In this case, the error bars were determined by converting the corresponding extreme values of stress, $\sigma_{f}\pm \Delta \sigma_{f}$, to strain (via Equation (7)), and then choosing the maximum absolute difference from $\epsilon_{f}$. These results are shown in Figure 6b, where again a linear fitting of the data is indicated with a dashed red (dotted blue) line for stress in the armchair (zigzag) direction. The linear fitting of the $\epsilon_{f}\left(T\right)$ data points leads to a slope $-4.7\times 10^{-3}$ % strain/K for stress in the armchair direction and $-1.6\times 10^{-2}$ % strain/K for stress in the zigzag direction. We can further compare these slopes to existing results by estimating the slopes of the failure strain versus temperature data reported in the literature. For uniaxial loading in the armchair direction, in Reference [9], a slope of $-4.8\times 10^{-3}$ % strain/K was estimated, which is in very good agreement with our result. A slope of $-5.4\times 10^{-3}$ % strain/K was estimated for the results presented in Reference [40], where, as noted above, the loading direction was unclear. Finally, the corresponding slope for strain applied in the armchair direction was $-5.9\times 10^{-3}$ % strain/K in Reference [14].

The fracture stress reported in the experimental study conducted in Reference [1] is 42±4 N/m. We obtained an intrinsic strength of 39.1±1.5 N/m (28.7±1.3 N/m) and a fracture strain of 18.5±1.5% (12.0±0.8%) for loading in the zigzag (armchair) direction at 300 K. We note that our results are in good agreement with the intrinsic strength and fracture strain values reported elsewhere, which additionally allow out-of-plane deformations of the material. Specifically, MD simulations carried out at 300 K using the AIREBO potential yield, for loading in the zigzag direction, intrinsic strengths of around 36−37 N/m and fracture strains between 17%and20% [3,5]. For loading in the armchair direction, Reference [47] conducted MD simulations using the REBO force field at 300 K, obtaining a fracture strain of 12.5% at a stress of 29.1 N/m, while Reference [5], who used AIREBO, reported an intrinsic strength of 30N/m and a fracture strain of 13%.

The results shown in Figure 6 indicate that graphene fails at lower applied stress/strain as temperature increases. This is reasonable since, as can be clearly seen from Figure 3, for the stress-controlled simulations considered here, the sheet achieves higher strains over the course of its oscillations by increasing temperature. As a result, for fixed stress, the bonds between neighboring atoms experience greater stretching at higher temperatures and therefore are more likely to break, causing failure of the material due to the increase in the maximum deformation of the lattice. Moreover, graphene can tolerate greater loads in the zigzag direction in the whole temperature range investigated here, as implied by the results in Figure 6, where the values of fracture strength and failure strain are consistently lower for stress in the armchair direction (red data points) as compared to stress applied in the zigzag direction (blue data points). In contrast to the relatively stronger temperature dependence of the fracture strength and failure strain, the Young’s modulus variation shown in Figure 5a exhibits a much smaller relative change, implying that the influence of thermal effects on the stiffness of graphene is less significant, at least within this temperature regime.

### 3.3. Bond Length and Bond Angle Distributions

In order to analyze the effects of temperature and stress on the distributions of the lengths and angles of the bonds, we first distinguish the two types of bond lengths, denoted by *A* and *Z*, and the two types of angles, indicated by α and ζ, as illustrated in Figure 1. The *A* bonds are in the armchair direction. The *Z* bonds alternate symmetrically in the zigzag direction and both exhibit identical deformations at 0 K when a uniaxial stress is applied in the high-symmetry zigzag or armchair directions. The angles α and ζ represent the bond angles formed between two consecutive *Z* bonds and between an *A* and a *Z* bond, respectively. They always respond oppositely under an applied stress due to the geometry of the system and the constraint α+2ζ=2π.

When a load is applied at zero temperature, due to the absence of fluctuations and the static nature of the strained sheet, there is no variability in the two types of bond lengths and angles, and their distribution is delta-peaked. Approximate expressions for the strain dependence of bond lengths *A* and *Z* and angles α and ζ were provided in Reference [42]. With the indices *a* or *z* used to indicate a load applied in the armchair or zigzag direction, respectively, these expressions read as follows:

(8) $$\begin{array}{cc} A_{a} & =1.42+0.011\;\epsilon_{0}+0.00024\;\epsilon_{0}^{2}, \end{array}$$

(9) $$\begin{array}{cc} Z_{a} & =1.42+0.0031\;\epsilon_{0}-0.000046\;\epsilon_{0}^{2}, \end{array}$$

and

(10) $$\begin{array}{cc} \alpha_{a} & =120^{\circ }-0.83\;\epsilon_{0}+0.020\;\epsilon_{0}^{2}, \end{array}$$

(11) $$\begin{array}{cc} \zeta_{a} & =120^{\circ }+0.41\;\epsilon_{0}-0.010\;\epsilon_{0}^{2}, \end{array}$$

while for stress in the zigzag direction,

(12) $$\begin{array}{cc} A_{z} & =1.42, \end{array}$$

(13) $$\begin{array}{cc} Z_{z} & =1.42+0.0088\;\epsilon_{0}+0.000080\;\epsilon_{0}^{2}, \end{array}$$

and

(14) $$\begin{array}{cc} \alpha_{z} & =120^{\circ }+0.80\;\epsilon_{0}-0.013\;\epsilon_{0}^{2} \end{array}$$

(15) $$\begin{array}{cc} \zeta_{z} & =120^{\circ }-0.40\;\epsilon_{0}+0.0064\;\epsilon_{0}^{2}. \end{array}$$

In Equations (8), (9), (12) and (13), the bond lengths *A* or *Z* are given in Å, and the zero-temperature strain $\epsilon_{0}$ is expressed as % strain. Similarly, in Equations (10), (11), (14) and (15), the bond angles α and ζ are provided in degrees, and $\epsilon_{0}$ should be given again as a percentage strain. The distributions of bond lengths (angles) in bulk graphene at T=0 K are given by double singular peaks at the locations provided by the above pairs of relations for the bond lengths (angles), depending on the direction of the loading for different values of the applied uniaxial strain.

In order to reveal the influence of temperature on the bond length and angle distributions, we registered all the fluctuating bond length and angle values during the system’s evolution in our measurement window and obtained normalized distributions for different amounts of stress/stain at various temperatures. In particular, we created a distribution for each realization by allocating all the measured bond lengths (angles) to fine-grained bins with a width of $3.5\times 10^{-3}$ Å ($0.004\;\frac{180^{\circ }}{\pi }$). The resulting distributions were normalized and then averaged over the different realizations in order to obtain the final distribution for each case. It is worth noting that the size of the error bars, indicating one standard deviation of this averaging computation over the different realizations, is negligible and hence not included in the plots of the distributions presented below. We emphasize again that we consider the central region of the sheet for collecting our data, as mentioned at the end of Section 3.1.

**Figure 7:**
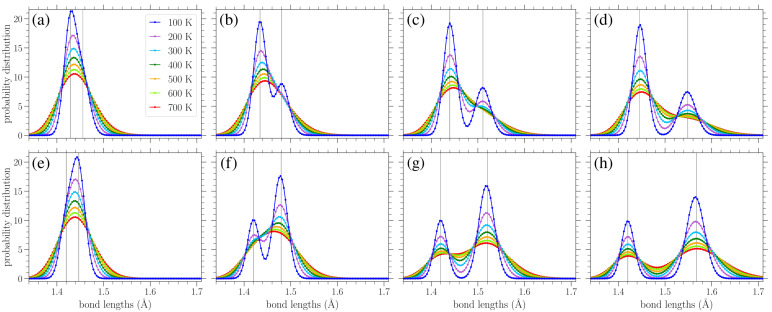
Normalized bond length distributions in graphene under increasing applied stress (left to right) in the armchair (top row) and zigzag (bottom row) directions at different temperatures *T*, as indicated in the legend. The curves are a guide for the eyes. The vertical lines indicate the values of the *A* and *Z* bond lengths at zero temperature based on Equations (8), (9), (12) and (13). The stresses σ in the armchair direction are (**a**) 0.569 eV/Å^2^, (**b**) 0.895 eV/Å^2^, (**c**) 1.22 eV/Å^2^, and (**d**) 1.55 eV/Å^2^, while those in the zigzag direction are (**e**) 0.563 eV/Å^2^, (**f**) 1.13 eV/Å^2^, (**g**) 1.69 eV/Å^2^, and (**h**) 2.16 eV/Å^2^.

In Figure 7a–d, we show the normalized bond length distributions for increasing values of stress applied in the armchair direction. In Figure 7e–h, the applied load is in the zigzag direction, and the stress increases from (e) to (h) too. When there is no loading, i.e., σ=0, at finite temperatures, the distributions are simply normal distributions with the variance linearly increasing with temperature (see Section 3.3.1). Increased temperature leads to larger fluctuations in the lattice, resulting in a wider spread of the observed bond lengths. In the presence of uniaxial loading, for the smaller values of stress presented in Figure 7a,e, there is a slight skewing of the distributions. As the stress is increased, the single peak splits into two peaks that are gradually separated more and more, as can be clearly seen from the plots corresponding to lower temperatures, due to the increased separation of the *A* and *Z* bond length values (see Figures 2 and 3 in Reference [42]). However, the increase in temperature leads to the merging of these two peaks due to their broadening. The centers of the peaks correspond to the zero-temperature values of the two types of bond lengths for each different direction of the applied stress, as given in Equations (8), (9), (12) and (13).

Since there are twice as many *Z*-type bond lengths as *A*-types, the highest peak in each distribution in Figure 7 mostly encompasses the lengths of the *Z*-type bonds. Thus, we can see that for stress applied in the armchair direction (Figure 7a–d), it is the *A* bonds that achieve greater lengths (the lower peak, further to the right), while the *Z* bonds exhibit smaller extension. In contrast, for stress applied in the zigzag direction (Figure 7e–h), the *Z* bonds achieve greater lengths (the taller peak is to the right in the distributions), while the centers of the smaller peaks remain near $r_{0}=1.42$ Å, in accordance with Equation (12) and the corresponding broadening due to thermal effects. The fact that all bonds stretch for stress applied in the armchair direction, but only the *Z* type bonds (two-thirds of all the considered bonds) are extended for a load in the other direction [42], proves that the gap between the two peaks is more pronounced for stress applied in the zigzag direction.

**Figure 8:**
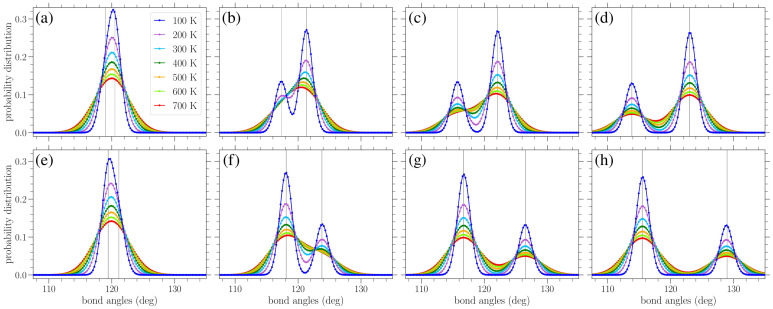
Normalized bond angle distributions in planar graphene under increasing stress (left to right) applied in the armchair (top row) and zigzag (bottom row) directions at various temperatures *T*, as indicated in the legend. The curves serve as a guide for the eyes. The vertical lines indicate the values of the α and ζ bond angles at zero temperature based on Equations (10), (11), (14) and (15). The values of stress σ in the armchair direction are (**a**) 0.244 eV/Å^2^, (**b**) 0.651 eV/Å^2^, (**c**) 1.06 eV/Å^2^, and (**d**) 1.55 eV/Å^2^, while those in the zigzag direction are (**e**) 0.282 eV/Å^2^, (**f**) 0.939 eV/Å^2^, (**g**) 1.60 eV/Å^2^, and (**h**) 2.16 eV/Å^2^.

**Figure 9:**
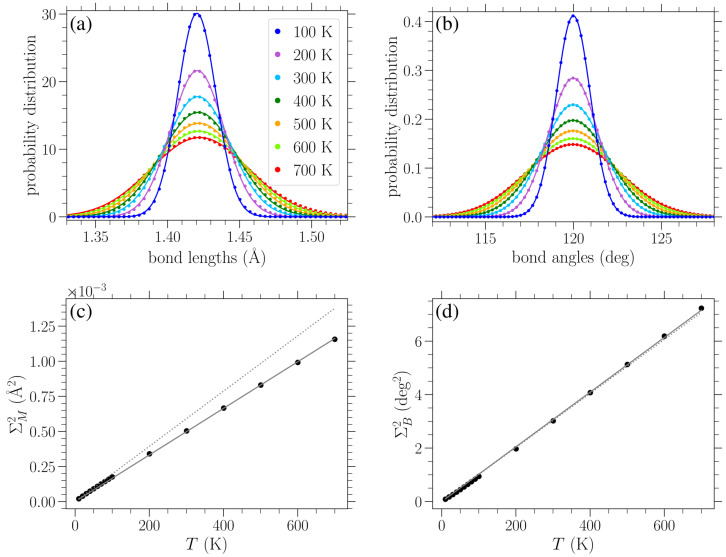
Normalized distributions of (**a**) bond lengths and (**b**) bond angles in bulk unstrained graphene (σ=0) at different temperatures *T*, as indicated in the legend, are indicated by circles. Solid lines in (**a**,**b**) represent Gaussian fittings of the numerical data. Circles represent temperature dependence in (**c**,**d**): the variance $\Sigma_{M}^{2}$ of the Gaussian fitting of the bond length distributions is presented in (**c**); the variance $\Sigma_{B}^{2}$ of the Gaussian fitting of the bond angle distributions is shown in (**d**). Solid lines in (**c**,**d**) indicate linear fittings of the corresponding data (see Equations (16) and (17)), while dotted lines denote the analytical approximate expressions of Equations (19) and (20), respectively.

In Figure 8, results similar to those given in Figure 7 are presented, but for the distribution of bond angles. At zero strain, normal distributions centered about the equilibrium angle of $\phi_{0}=120^{\circ }$ were obtained for finite temperatures, with a variance increasing with temperature (see Figure 9b below). Again, we see the gradual peak splitting due to increased stress, while increasing the temperature leads to the broadening and merging of these peaks. The highest peak in the bond angle distributions corresponds to the ζ-type angles, since there are twice as many ζ angles as α angles. For stress in the armchair direction (Figure 8a–d), the α angles decrease, while the ζ angles increase. The reverse is true when the stress is applied in the zigzag direction (Figure 8e–h). Also, in this case, the peaks of the distributions are centered about the zero-temperature α and ζ values, as given in Equations (10) and (11) or (14) and (15), depending on the direction of the applied stress.

#### 3.3.1. Analytical Expressions for the Bond Length and Bond Angle Distributions

We now present approximate analytical expressions for the bond length and angle distributions, as shown in Figure 7 and Figure 8, in order to describe the dependence of graphene’s structural properties on stress and temperature. Based on the results discussed in the previous subsection, we note that the obtained distributions appear to approximately be given through the combination of two normal distributions, where the means of these normal distributions correspond to the values of the two types of bond lengths, or angles, found for each stress at zero temperature. The variance of these normal distributions is induced by thermal fluctuations, while the difference in peak heights is related to the fact that there exist twice as many of one type of bond length (or angle) as the other.

As there exist approximate expressions for the equilibrium bond lengths and bond angles as a function of the applied strain at T=0 K (see Equations (8)–(15)), the explicit dependence of variance on temperature remains to be determined. This will be achieved by numerically evaluating the effects of temperature on the normal distributions of the bond lengths and bond angles for the unstrained graphene sheet. The results of these calculations will be compared with analytical estimates of the variance through the Boltzmann distribution, using a second-order approximation on the relevant potential energy terms describing bond stretching and angle bending.

Performing a Gaussian-curve-fitting procedure for the numerically obtained distributions of the bond lengths and bond angles at zero applied stress for various temperatures (as shown in Figure 9a,b, respectively), we computed the corresponding variances and mean values. The dependence of these variances on temperature is indicated by filled circles in Figure 9c,d for the bond length and angle distributions, respectively. Solid lines in the remaining plots denote a linear fitting of the data. It is worth noting that the mean of the bond length distribution slightly increases with temperature too due to the soft Morse potential describing bond stretching. However, incorporating this small variation in the mean value with temperature does not practically affect the results discussed below. The mean of the bond angle distribution does not change with temperature, as expected due to the equality of the α and ζ angles in the unstrained graphene and their constrained sum.

A linear fitting describes the dependence of the variance $\Sigma_{M}^{2}$ of bond length distributions on temperature *T*:

(16) $$\Sigma_{M}^{2}\left(T\right)=C_{M}\;T,$$

with $C_{M}=1.66\times 10^{-6}$ Å^2^/K (solid line in Figure 9c). Similarly, the numerically found variances $\Sigma_{B}^{2}$ for the bond angle distributions are well described by

(17) $$\Sigma_{B}^{2}\left(T\right)=C_{B}\;T,$$

with $C_{B}=1.02\times 10^{-2}$ deg^2^/K (solid line in Figure 9d).

The proportionality of these variances with temperature can be derived through the Boltzmann distribution when a quadratic approximation of the corresponding potential energy is considered. In particular, according to the second derivative of the Morse potential of Equation (1),

(18) $$V_{M}^{\prime \prime }\left(r\right)=-2Da^{2}e^{-2a(r-r_{0})}\left(e^{a(r-r_{0})}-2\right),$$

the second order approximation of the bond-stretching energy term about the equilibrium $r=r_{0}$ reads $V_{M}^{lin}=a^{2}D{\left(r-r_{0}\right)}^{2}$. Using this approximation, the corresponding Boltzmann distribution $\exp\left(\frac{-a^{2}D{\left(r-r_{0}\right)}^{2}}{k_{B}T}\right)$ results in a normal bond length distribution of the form $\exp\left(\frac{-{\left(r-r_{0}\right)}^{2}}{2\Sigma_{M}^{2}}\right)$ centered about the mean $r_{0}$ with a variance

(19) $$\Sigma_{M}^{2}\left(T\right)=\frac{k_{B}}{2a^{2}D}\;T,$$

which gives $\Sigma_{M}^{2}\left(T\right)=1.97\times 10^{-6}$ (Å^2^/K) ·T when the parameters of the Morse potential are substituted.

Following a similar approach to estimate the variance of the bond angle distributions for different temperatures, we consider the second-order approximation of the potential $V_{B}$, Equation (2), about $\phi =\phi_{0}=120^{\circ }$, given by $V_{B}^{lin}=\frac{d}{2}\frac{\pi^{2}}{180^{2}}{\left(\phi -\phi_{0}\right)}^{2}$ (when angles are measured in degrees). Note that due to the constraints on the sums of the bond angles around a particular atom and on the sums within hexagonal rings, just one angle can not be varied alone. When an angle slightly varies from the equilibrium value, at least three other angles should also change. Thus, multiplying the linearized angle-bending energy in the Boltzmann distribution by a factor 4, we eventually find the variance (in squared degrees), about the mean $\phi_{0}$, of the bond angle distribution

(20) $$\Sigma_{B}^{2}\left(T\right)=\frac{k_{B}180^{2}}{4\pi^{2}d}\;T,$$

which results in $\Sigma_{B}^{2}\left(T\right)=1.01\times 10^{-2}$ (deg^2^/K) ·T, using the value of *d*.

The dotted lines in Figure 9c,d correspond to the analytical expressions of Equations (19) and (20), respectively. We can see from Figure 9c that the analytically obtained slope of Equation (19) is somehow larger than the corresponding numerical value of Equation (16) (the relative difference is less than 20%). Concerning the variance of the bond angle distributions, Figure 9d shows an excellent agreement between the analytically and numerically obtained slopes, exhibiting a relative difference of less than 1%. One reason for the quantitative disparity between the analytical prediction and numerical determination of the slope of the linear temperature dependence of the variance of the bond length distributions, but not for the angle distributions, may be the fact that the second-order approximation of the angle-bending potential $V_{B}\left(\phi \right)$ of Equation (2) is valid for a wide range of angles (see Figure 2 in Reference [12]). However, due to the highly anharmonic nature of the Morse potential $V_{M}\left(r\right)$, Equation (1), at the same energy scales (see Figure 1 in Reference [12]), the second-order approximation about $r_{0}$ is only valid when very close to $r_{0}$.

By combining the numerically determined variances for different temperatures and the known bond length and angle mean values as a function of the applied stress/strain, analytical approximate expressions for the bond length and angle distributions can be derived. Regarding the bond length distributions, an additional issue should be taken into account when the numerically determined variances from Equation (16) will be used. In particular, the relation between variance and temperature should be scaled according to the behavior of $V_{M}^{\prime \prime }$ (Equation (18)) at the mean of the corresponding peak of the distribution since bond lengths even further away from $r_{0}$ are encountered once stress is applied to the system and the second derivative of the Morse potential varies significantly with *r*. Given that, analytically, the variance equates to $k_{B}T/V_{M}^{\prime \prime }\left(r_{0}\right)$, when close to $r=r_{0}$, we multiply the numerically determined variance from Equation (16) by the scaling function

(21) $$F\left(r\right)=\frac{V_{M}^{\prime \prime }\left(r_{0}\right)}{V_{M}^{\prime \prime }\left(r\right)},$$

where *r* is the known mean of the peak of interest in the distribution, provided by either Equations (8) and (9) or Equations (12) and (13) depending on the loading direction.

As a result, the bond length distribution for a given applied stress/strain and temperature *T* can be approximated by the relation

(22) $$P_{M}=\frac{1}{3\sqrt{2\pi \;C_{M}F\left(A\right)\;T}}\exp\left(-\frac{{\left(r-A\right)}^{2}}{2\;C_{M}F\left(A\right)\;T}\right)+\frac{2}{3\sqrt{2\pi \;C_{M}F\left(Z\right)\;T}}\exp\left(-\frac{{\left(r-Z\right)}^{2}}{2\;C_{M}F\left(Z\right)\;T}\right),$$

where *A* and *Z* are functions of the applied stress/strain, determined in Equations (8) and (9) or Equations (12) and (13), for stress in the armchair or zigzag direction, respectively, $C_{M}$ is given in Equation (16), and F(r) is yielded by Equation (21). The factor of 2 in the second term is present because there are twice as many *Z* bonds as *A* bonds. Division by 3 is employed for normalizing the distribution. Note that the quantities *A* and *Z* are provided by the corresponding zero-temperature relations in Equations (8), (9), (12) and (13) as a function of strain ϵ. If they are needed as a function of stress σ, the stress–strain relation of Equation (7) should be used to change the variable of the applied load.

For the bond angle distributions, the subtlety mentioned above concerning the scaling function is not needed since the second derivative of the angle-bending potential, Equation (2), is the same everywhere regardless of the angle value at the peak of the distribution. Therefore, the angle-bending distributions can be approximated by the expression

(23) $$P_{B}=\frac{1}{3\sqrt{2\pi \;C_{B}\;T}}\left[\exp\left(-\frac{{\left(\phi -\alpha \right)}^{2}}{2\;C_{B}\;T}\right)+2\;\exp\left(-\frac{{\left(\phi -\zeta \right)}^{2}}{2\;C_{B}\;T}\right)\right],$$

where α and ζ are determined by the applied stress/strain from Equations (10) and (11) or (14) and (15), depending on the loading direction, and $C_{B}$ is provided by Equation (17). The factor 2 in the second term is present because there are twice as many ζ angles as α angles, and division by 3 normalizes the distribution. If the loading is given through the value of stress, Equation (7) can be also used.

**Figure 10:**
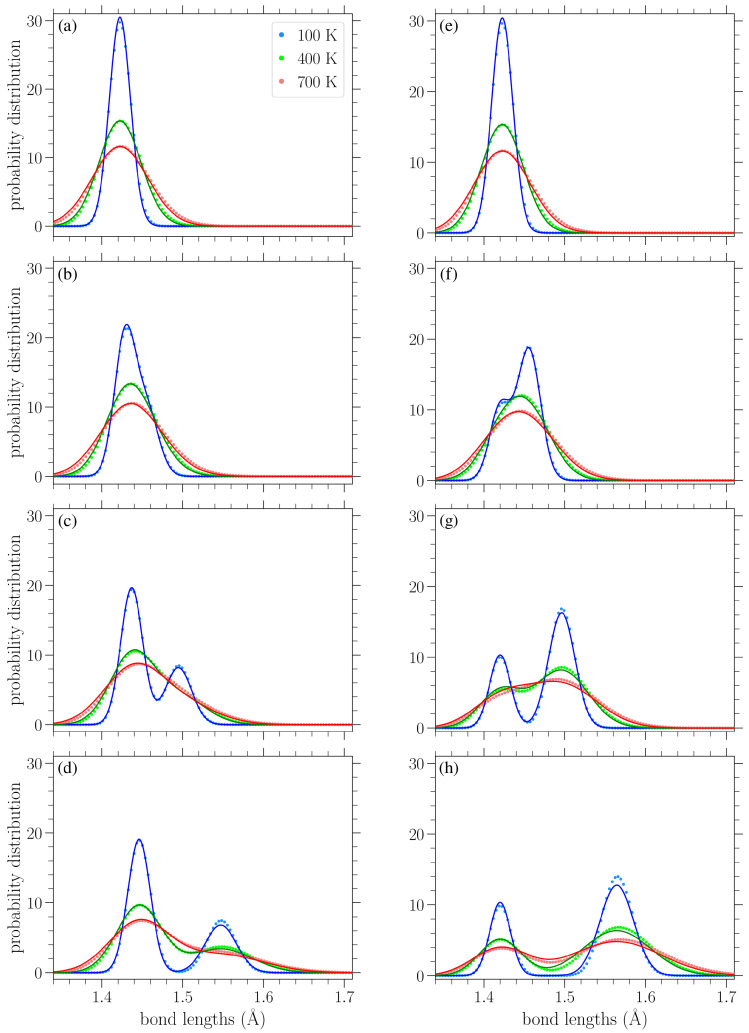
Bond length distributions at various temperatures *T* (as shown in the legend) and under various applied stresses in the armchair direction (left column) or the zigzag direction (right column) under increasing loads from top to bottom. In particular, the stresses σ in the armchair direction are (**a**) 0.0813 eV/Å^2^, (**b**) 0.569 eV/Å^2^, (**c**) 1.06 eV/Å^2^, and (**d**) 1.55 eV/Å^2^. The stresses in the zigzag direction are (**e**) 0.0939 eV/Å^2^, (**f**) 0.751 eV/Å^2^, (**g**) 1.41 eV/Å^2^, and (**h**) 2.16 eV/Å^2^. The analytical expressions of Equation (22) are indicated by solid curves, and the corresponding numerical data are indicated by circles.

**Figure 11:**
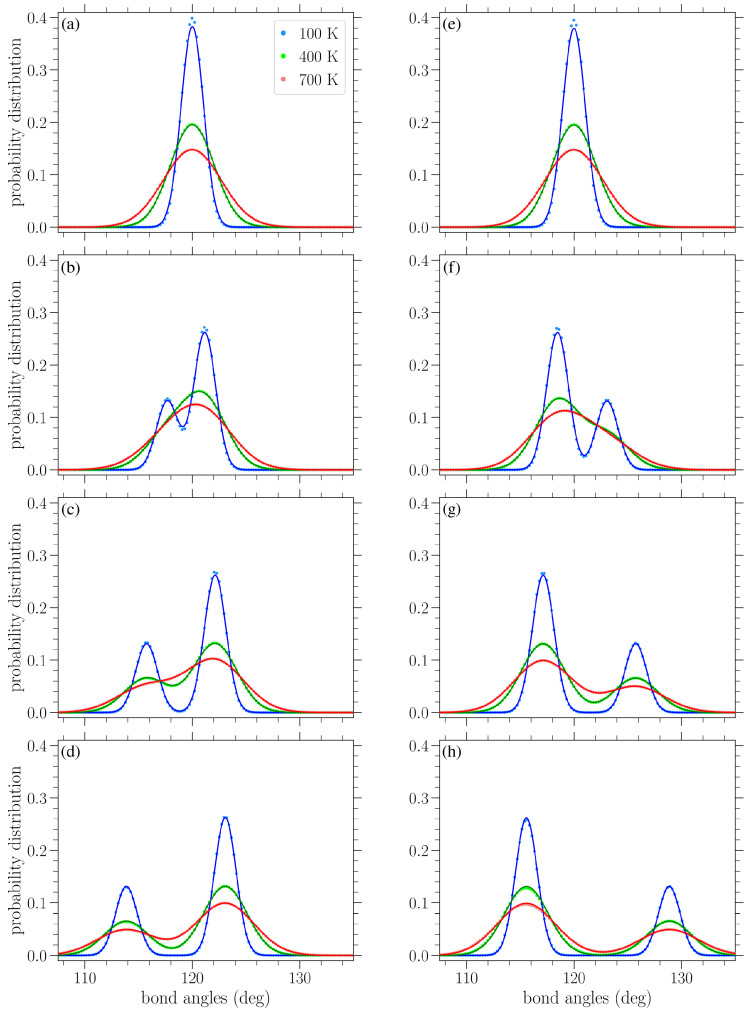
Bond angle distributions for different temperatures *T* (as shown in the legend) and stresses in the armchair direction (left column) or the zigzag direction (right column) under increasing values of stress from top to bottom. The stress in the armchair direction is (**a**) 0.0813 eV/Å^2^, (**b**) 0.569 eV/Å^2^, (**c**) 1.06 eV/Å^2^, and (**d**) 1.55 eV/Å^2^, while that in the zigzag direction is (**e**) 0.0939 eV/Å^2^, (**f**) 0.751 eV/Å^2^, (**g**) 1.41 eV/Å^2^, and (**h**) 2.16 eV/Å^2^. The analytical expressions given by Equation (23) are indicated by the solid curves, and the corresponding numerical data are indicated by circles.

The circles in Figure 10 present the numerically computed bond length distributions at various applied stresses and temperatures, while the solid lines correspond to the curve $P_{M}$ from Equation (22). Figure 11 contains similar results, but for the bond angle distributions. These plots show that the analytical expressions presented above provide a reasonable description of the bond length and angle distributions in strained graphene at various temperatures, at least up to the values considered here. In Figure 10, at the larger values of applied stress and lower temperatures, the analytical distribution $P_{M}$ of Equation (22) underestimates the longer-bond (second) peak of the numerically obtained distributions (Figure 10d,g,h). Figure 10h shows the greatest deviation of the analytical expression from the numerical data at the right-hand peak of the lowest temperature at T=100 K; in this case, the difference is 8.7%. From the plots of Figure 11, we can see that the analytical expression $P_{B}$ (Equation (23)) describes the data quite well, apart from small discrepancies at the heights of the taller peak at the lower temperatures depicted and for the smaller values of stress. Figure 11a shows the biggest deviation for the T=100 K case, where the value of the analytical expression is 4.1% below that of the numerical data. In any case, both expressions of Equations (22) and (23) provide a useful analytical description of the underlying structural properties of the strained graphene at finite temperatures.

## 4. Conclusions

We investigated the planar dynamics of a uniaxially loaded graphene sheet using Hamiltonian formalism and an efficient symplectic integration technique allowing the collection of accurate numerical data for very long simulation times. Our MD simulations examined the effects of thermal fluctuations in the mechanical response of graphene. In particular, we derived stress–strain responses for two different directions of applied stress, namely, along either the armchair or the zigzag direction, at various temperatures.

A small, almost linear decrease in the Young’s modulus of graphene was obtained as the temperature of the sheet increased. Such a variation in the Young’s modulus with temperature is in line with previous investigations. Furthermore, an intriguing temperature dependence of the third-order elastic modulus has been presented for the first time; this dependence was found to decrease (slightly increase) its absolute value with an increasing temperature for stresses in the armchair (zigzag) direction. Finally, we found that tensile strength and failure strain decrease approximately linearly with temperature and computed the slope of this variation. A quantitative comparison with existing results regarding these variations was presented.

It is worth mentioning that even though our model is restricted to planar deformations, the results obtained for the intrinsic strength and fracture strain at room temperature are in agreement with MD simulations [3,5,47], allowing out-of-plane displacements of carbon atoms. Moreover, the values of Young’s modulus and fracture strength at 300 K are in accordance with the experimental estimates presented in References [1,2,3] and Reference [1], respectively.

The dependence of the distributions of bond lengths and bond angles within the graphene sheet on both the applied stress and temperature has also been discussed. Approximate analytical expressions for these distributions were provided. In particular, we found that the distributions can be described by the sum of two Gaussian peaks, where the center of each peak is obtained from the values of bond lengths or bond angles, respectively, in the strained graphene subjected to a particular amount of stress at zero temperature. The variance of each peak as a function of temperature was derived by employing the corresponding data at zero applied stress, while for the bond length distributions, a scaling factor was additionally incorporated to account for the anharmonicity of the Morse potential. Thus, for the first time, a detailed description of the effects of both stress and temperature on the structural properties of graphene is reported.

## Data Availability

The raw data supporting the conclusions of this article will be made available by the authors on request.
